# Interleukin-34 as a fibroblast-derived marker of liver fibrosis in patients with non-alcoholic fatty liver disease

**DOI:** 10.1038/srep28814

**Published:** 2016-07-01

**Authors:** Hirotaka Shoji, Sachiyo Yoshio, Yohei Mano, Erina Kumagai, Masaya Sugiyama, Masaaki Korenaga, Taeang Arai, Norio Itokawa, Masanori Atsukawa, Hiroshi Aikata, Hideyuki Hyogo, Kazuaki Chayama, Tomohiko Ohashi, Kiyoaki Ito, Masashi Yoneda, Yuichi Nozaki, Takumi Kawaguchi, Takuji Torimura, Masanori Abe, Yoichi Hiasa, Moto Fukai, Toshiya Kamiyama, Akinobu Taketomi, Masashi Mizokami, Tatsuya Kanto

**Affiliations:** 1 The Research Center for Hepatitis and Immunology, National Center for Global Health and Medicine, Ichikawa, Chiba, Japan; 2 Department of Gastroenterological Surgery I, Hokkaido University Graduate School of Medicine, Sapporo, Hokkaido, Japan; 3 Division of Gastroenterology, Department of Internal Medicine, Nippon Medical School Chiba Hokusoh Hospital, Inzai, Chiba, Japan; 4 Department of Gastroenterology and Metabolism, Applied Life Sciences, Institute of Biomedical & Health Sciences, Hiroshima University, Hiroshima, Hiroshima, Japan; 5 Department of Gastroenterology and Hepatology, JA Hiroshima General Hospital, Hatsukaichi, Hiroshima, Japan; 6 Division of Gastroenterology, Department of Internal Medicine, Aichi Medical University School of Medicine, Nagakute, Aichi, Japan; 7 Department of Gastroenterology, National Center for Global Health and Medicine, Shinjuku, Tokyo, Japan; 8 Division of Gastroenterology, Department of Medicine, Kurume University School of Medicine, Kurume, Fukuoka, Japan; 9 Department of Gastroenterology and Metabology, Ehime University Graduate School of Medicine, Toon, Ehime, Japan

## Abstract

Non-alcoholic fatty liver disease (NAFLD) is a common cause of chronic non-viral liver disease. Activation of macrophages and hepatic stellate cells is a critical step that promotes liver fibrosis. We aimed to explore the feasibility of interleukin-34 (IL-34), a key regulator of macrophages, as a fibrosis marker in patients with NAFLD. We enrolled 197 liver biopsy-proven NAFLD patients. We evaluated the serum levels of IL-34, macrophage-colony stimulating factor (M-CSF), soluble CD163 (sCD163), 40 cytokines/chemokines, hyaluronic acid, type IV collagen 7s, and clinically-approved fibrosis scores. IL-34 increased with the progression of fibrosis and was an independent marker for liver fibrosis. Immunostaining experiments, using resected liver specimens from NAFLD patients, revealed that IL-34 was mainly expressed on liver fibroblasts. IL-34 based fibrosis score (0.0387*IL-34 (pg/ml) + 0.3623*type IV collagen 7s (ng/ml) + 0.0184*age (year)–1.1850) was a practical predictive model of liver fibrosis. Using receiver-operating characteristic analyses, the area under the curve, sensitivity, and specificity of IL-34 based fibrosis score were superior or comparable to the other fibrosis biomarkers and scores. In conclusion, the IL-34 based fibrosis score, including serum IL-34, type IV collagen 7s and age, is a feasible diagnostic marker of liver fibrosis in NAFLD patients.

Non-alcoholic fatty liver disease (NAFLD) is one of the most common causes of chronic liver disease, with a 15–30% prevalence rate worldwide^1^,^2^. NAFLD is regarded as a consequence of metabolic syndrome, often accompanied by cardiovascular disease, diabetes, and/or dyslipidemia^3^,^4^,^5^. NAFLD is a progressive liver disease, evolving from simple steatosis, non-alcoholic steatohepatitis (NASH), liver cirrhosis to hepatocellular carcinoma (HCC)^6^,^7^. About 25% of patients with simple steatosis progress to the more serious stage of NASH, and 20% of patients with NASH progress to liver cirrhosis or HCC over 20–30 years^8^,^9^. The degree of fibrosis is one of the critical features that indicates the prognosis of patients with NAFLD, and thus evaluating the fibrosis stage is crucial for the management of patients with NAFLD.

Liver biopsy still remains the gold standard for evaluating the degree of hepatic necro-inflammation and fibrosis in patients with chronic liver disease^10^. However, there are substantial limitations with respect to liver biopsy: serious complications are sometimes unavoidable, including bleeding or biliary tract infection^11^,^12^. Furthermore, repetitive biopsy for sequential assessment of the disease is not possible in actual clinical practice. Alternatively, several clinical biomarkers, non-invasive indices, and ultrasound-based imaging techniques have been developed, the problems of which are complexity, reproducibility, the influence of ethnic constitutional differences and costs for routine clinical use^13^,^14^.

Clinical and basic researchers have been focusing on the mechanisms of liver fibrosis. “Multiple hits” including genetic and environmental factors are required for the progression of fibrosis^9^. Macrophages, as an activator of hepatic stellate cells (HSCs), have been reported to play essential roles in liver fibrogenesis^15^,^16^,^17^. Interleukin-34 (IL-34), a ligand for colony-stimulating factor-1 receptor (CSF-1R)^18^, like macrophage colony-stimulating factor (M-CSF), binds to CSF-1R and promotes differentiation, proliferation and survival of monocytes and macrophages^19^. Preisser *et al*. recently reported that IL-34 and M-CSF increased in the sera of hepatitis C virus (HCV) infected patients with advanced liver fibrosis and that they were capable of inducing pro-fibrotic macrophages *in vitro*^20^. Kazankov *et al*. reported that soluble CD163 (sCD163) levels increased in association with the fibrosis stage in patients with chronic hepatitis C, suggesting that sCD163 can be useful as a marker of liver fibrosis^21^. CD163 is expressed on activated macrophages, and its soluble form is shed from the macrophages and released into the blood^22^,^23^.

In this study, we evaluated the possibility of using the above-mentioned macrophage-related factors as a marker of liver fibrosis in patients with NAFLD. We found that IL-34 increased with the progression of liver fibrosis and that IL-34 could serve as an independent marker of liver fibrosis. Furthermore, an index consisting of serum IL-34, type IV collagen 7s, and age, which was established by multiple regression analyses, was superior to IL-34, M-CSF, sCD163, macrophage inflammatory protein-3 alpha/C-C motif chemokine ligand 20 (MIP-3α/CCL20), hyaluronic acid, type IV collagen 7s, and fibrosis scores in diagnosing the liver-fibrosis stages. We also showed that liver fibroblasts are the main producer of IL-34 in patients with NAFLD.

## Results

### Serum IL-34 in patients with NAFLD increased with the progression of fibrosis

**Figure 1:**
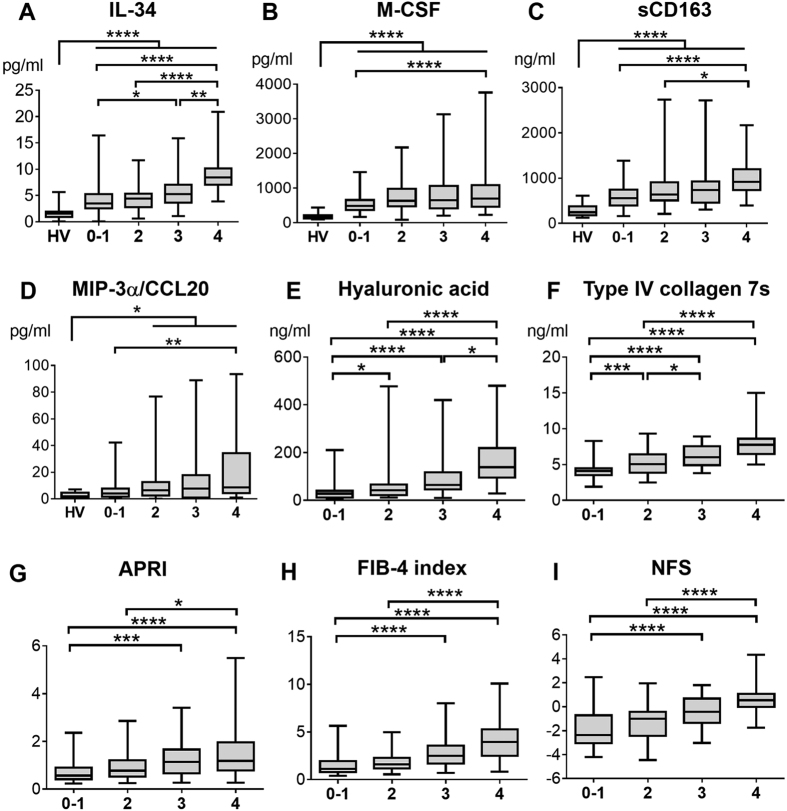
Serum IL-34, M-CSF, sCD163, MIP-3α/CCL20, hyaluronic acid, type IV collagen 7s, APRI, FIB-4 index, and NFS increase of NAFLD patients with progression of fibrosis. Serum interleukin-34 (IL-34), other biomarkers and fibrosis scores are shown for patients with non-alcoholic fatty liver disease (NAFLD) stratified by the stage of fibrosis (Brunt’s criteria). IL-34 (**A**), macrophage-colony stimulating factor (M-CSF) (**B**), soluble CD163 (sCD163) (**C**), macrophage inflammatory protein-3 alpha/C-C motif chemokine ligand 20 (MIP-3α/CCL20) (**D**), hyaluronic acid (**E**), type IV collagen 7s (**F**), aspartate transaminase (AST)-to-platelet ratio index (APRI) (**G**), FIB-4 index (**H**), and NAFLD fibrosis score (NFS) (**I**). Box plots represent the interquartile range and the whiskers show the minimum and maximum values. The line of each box shows the median. *p < 0.05, **p < 0.01, ***p < 0.001, ****p < 0.0001 by Kruskal-Wallis test with Dunn’s multiple comparison test HV, healthy volunteer; 0–1, Stage 0–1 (Brunt’s criteria); 2, Stage 2; 3, Stage 3, 4, Stage 4.

**Figure 2:**
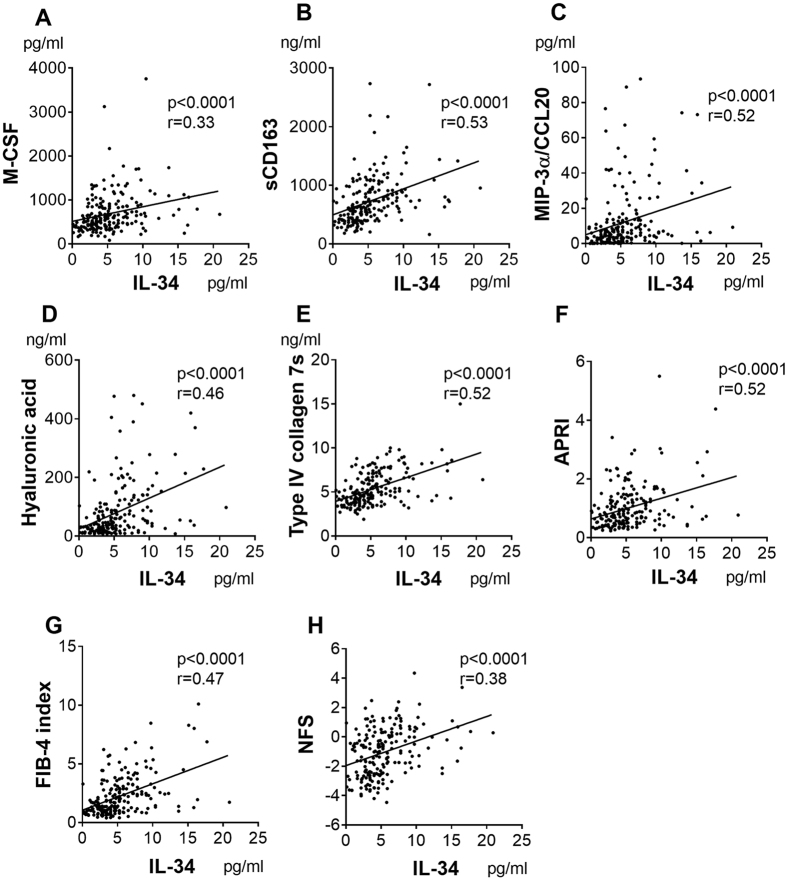
Serum IL-34 correlates with M-CSF, sCD163, MIP-3α/CCL20, hyaluronic acid, type IV collagen 7s, APRI, FIB-4 index, and NFS of patients with NAFLD. The correlations between interleukin-34 (IL-34) and each biomarker, clinical parameter and fibrosis score were assessed by Spearman’s rank-correlation coefficient. The p-values and correlation coefficients are shown in each plot. macrophage-colony stimulating factor (M-CSF) (**A**), soluble CD163 (sCD163) (**B**), macrophage inflammatory protein-3 alpha/C-C motif chemokine ligand 20 (MIP-3α/CCL20) (**C**), hyaluronic acid (**D**), type IV collagen 7s (**E**), aspartate transaminase (AST)-to-platelet ratio index (APRI) (**F**), FIB-4 index (**G**), and NAFLD fibrosis score (NFS) **(H**).

Because we hypothesized that macrophage-related factors could serve as biomarkers of fibrosis, we examined serum levels of IL-34, M-CSF, and sCD163 as well as hyaluronic acid, type IV collagen 7s, and aspartate transaminase (AST)-to-platelet ratio index (APRI), FIB-4 index, and NAFLD fibrosis score (NFS), in 197 liver biopsy-proven NAFLD patients and 20 healthy volunteers (HVs). The clinical backgrounds of these patients are shown in Table 1. We also evaluated an additional 40 cytokines/chemokines for the purpose of exploring new factors associated with fibrosis in NAFLD patients (Supplementary Table S1). Serum IL-34, M-CSF, sCD163, and MIP-3α/CCL20 increased in NAFLD patients with the progression of fibrosis (Fig. 1A–D). Similar results were obtained for hyaluronic acid, type IV collagen 7s, APRI, FIB-4 index, and NFS (Fig. 1E–I). Serum IL-34 levels were positively correlated with serum M-CSF, sCD163, MIP-3α/CCL20, hyaluronic acid, or type IV collagen 7s levels and with APRI, FIB-4 index, or NFS (Fig. 2). As previously reported^24^,^25^, the serum levels of tumor necrosis factor alpha (TNF-α) and interleukin-1β (IL-1β) were elevated in NAFLD patients regardless of fibrosis stages (data not shown), suggesting that these factors are related to the presence of fatty liver disease. From these data, we aimed to investigate the possibility of IL-34, M-CSF, sCD163, MIP-3α/CCL20, hyaluronic acid, type IV collagen 7s, APRI, FIB-4 index, and NFS as a marker of liver fibrosis in NAFLD patients.

### Serum IL-34 was an independent marker of liver fibrosis in NAFLD patients

In univariate analyses, IL-34, M-CSF, sCD163, hyaluronic acid, type IV collagen 7s, APRI, FIB-4 index, and NFS were associated with liver fibrosis (Table 2 and Supplementary Table S2–3) in each stage. Furthermore, age, platelet counts, prothrombin time, cholesterol amount, and albumin level were also relevant to liver cirrhosis (Stage 4) (Table 2). In multivariate analyses, IL-34 was an independent marker for liver cirrhosis (Stage 4) [odds ratio (OR) = 1.233, p = 0.006] (Table 2). Type IV collagen 7s was independently associated with liver fibrosis among all stages [significant fibrosis (≥Stage 2), OR = 2.259, p < 0.001; advanced fibrosis (≥Stage 3), OR = 1.940, p < 0.001; liver cirrhosis (Stage 4), OR = 1.910, p = 0.002] (Table 2 and Supplementary Table S2–3).

Using receiver-operating characteristic (ROC) analyses, the area under the curves (AUC) of IL-34 were 0.68 for significant fibrosis (≥Stage 2), 0.76 for advanced fibrosis (≥Stage 3), and 0.87 for liver cirrhosis (Stage 4) (Table 3). For the diagnosis of liver cirrhosis (Stage 4), the AUC, sensitivity, and specificity of IL-34, which were 0.87, 83.3%, and 80.2%, respectively, were superior to or comparable with the other serum biomarkers and fibrosis indexes (Table 3).

Serum IL-34 levels, as well as M-CSF, sCD163, APRI, and FIB-4 index, were also elevated in HCV-positive patients with advanced fibrosis/cirrhosis (≥Stage 3) (Supplementary Table S4, Supplementary Fig. S1A–E). Using ROC analyses for the diagnosis of advanced fibrosis (≥Stage 3), AUC of IL-34 was 0.95 (Supplementary Fig. S1F–G and Supplementary Table S5). The AUC, sensitivity, and specificity of IL-34, which were 0.95, 100%, and 83.9%, were superior to or comparable with M-CSF, sCD163, APRI, and FIB-4 index (Supplementary Table S5). These results show that serum IL-34 can serve as a feasible diagnostic marker of fibrosis not only in patients with NAFLD but also in those with chronic HCV infection.

### IL-34 was mainly expressed on liver fibroblasts

**Figure 3:**
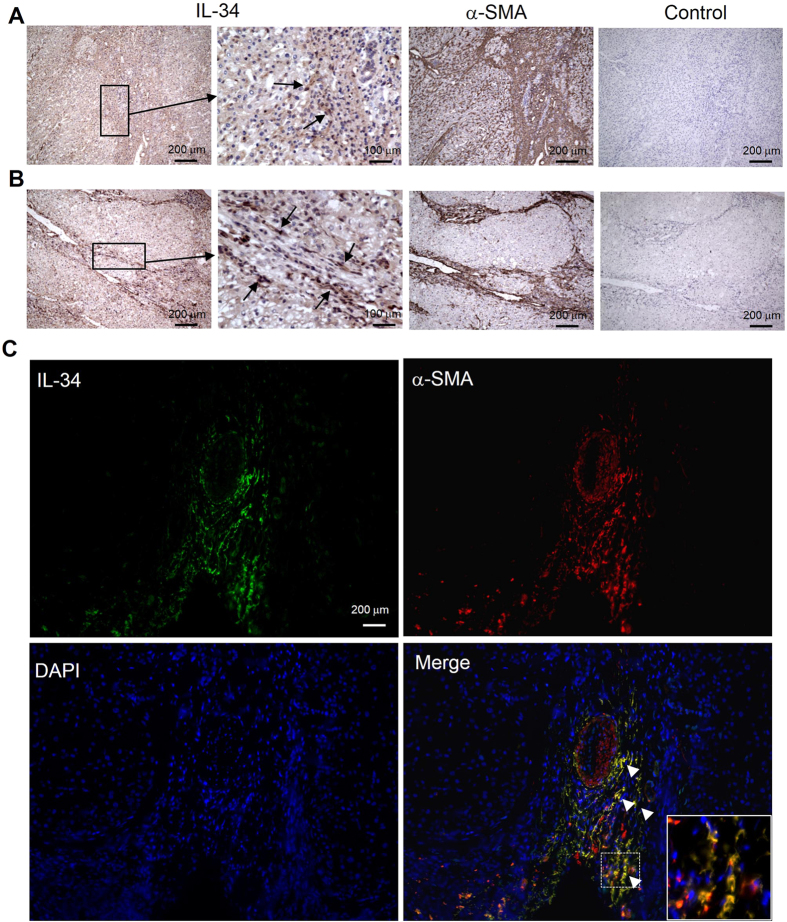
Liver fibroblasts express IL-34 in NAFLD patients. Liver specimens were obtained from patients with non-alcoholic fatty liver disease (NAFLD) (liver cirrhosis/Stage 4 (**A**) and advanced fibrosis/Stage 3 (**B**). Serial sections from these resected liver specimens were labeled as follows: mouse anti-human interleukin-34 (IL-34) monoclonal and secondary goat anti-mouse antibody (Ab) (IL-34 staining), mouse anti-human alpha smooth muscle actin (α–SMA) Ab and secondary goat anti-mouse Ab (α-SMA staining), secondary goat anti-mouse Ab (negative control for IL-34 staining). Arrows in the (**A**,**B**) panels indicate the fibroblasts expressing IL-34. Immunofluorescence staining on frozen liver specimens from patients with NAFLD was shown (**C**) (x200, green, IL-34; red, α-SMA; blue, DAPI). White arrowheads mark the positions of IL-34 and α-SMA positive cells. Inset of the photomicrograph shows fluorescent merged cells in enlarged scale.

We stained the non-cancerous liver specimens obtained from NAFLD patients with immunohistochemistry. The serial liver sections were stained with anti-IL-34 antibody (Ab) and anti-alpha smooth muscle actin (α-SMA) Ab. IL-34 was expressed mainly on the fibroblasts identified as α–SMA positive [liver cirrhosis (Stage 4) (Fig. 3A) and advanced fibrosis (Stage 3) (Fig. 3B)]. In order to investigate precisely the source of IL-34 in liver tissue, we stained liver specimens with a combination of immunofluorescence Abs. Almost all of the IL-34 positive cells were fibroblasts (α-SMA-positive cells) (Fig. 3C). IL 34 was not expressed on Kupffer cells (CD68-positive cells) (Supplementary Fig. S2). Primary fibroblasts obtained from surgically resected liver tissues from NAFLD patients also expressed IL-34 (Supplementary Fig. S3A,B). Such primary fibroblasts expressed IL-34 more than LX-2 (hepatic stellate cell line) on a per cell basis (Supplementary Fig. S3C,D). Furthermore, the expression levels of IL-34 in primary fibroblasts significantly increased in response to TNF-α (Supplementary Fig. S3E). We also stained the non-cancerous liver specimens obtained from patients with HCV infection, and IL-34 was expressed mainly on the fibroblasts [liver cirrhosis/Stage 4 (Supplementary Fig. S4)]. These results suggest that the elevation of serum IL-34 in patients with liver fibrosis might be due to the increased fibroblasts and their enhanced expression of IL-34 in the liver.

### The predictive model based on serum IL-34, type IV collagen 7s, and age was a practical diagnostic method of liver fibrosis in NAFLD patients

**Figure 4:**
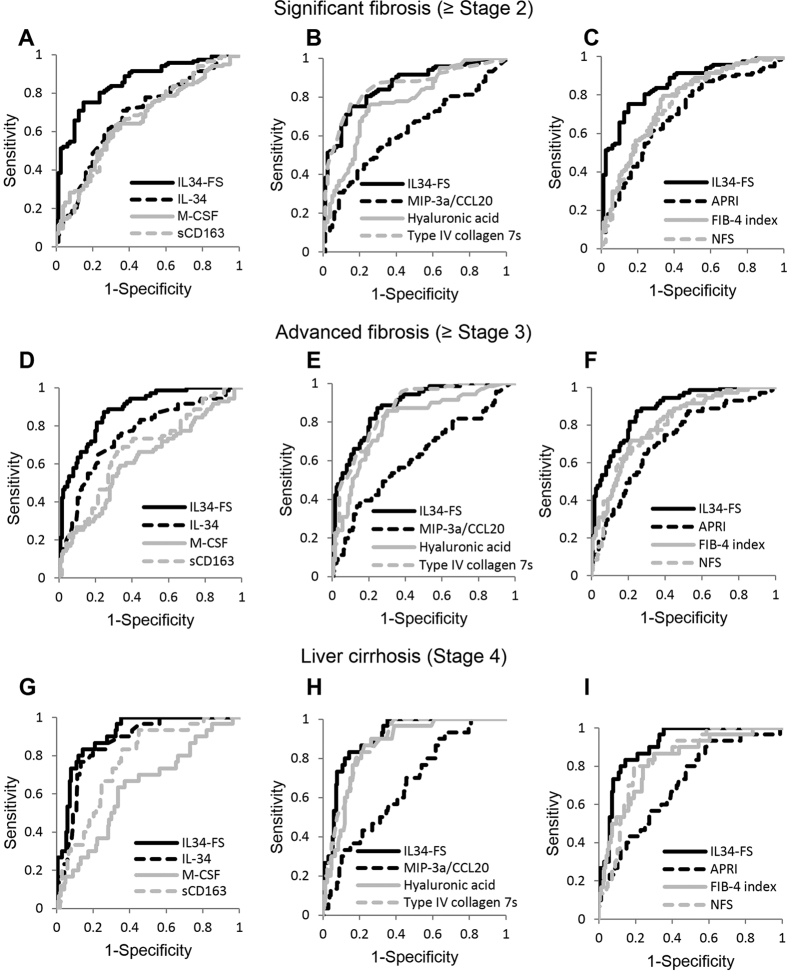
ROC analyses of IL34-FS, IL-34, other biomarkers, and fibrosis scores for diagnosis of each stage of patients with NAFLD. Receiver-operating characteristic (ROC) curves of interleukin-34 based fibrosis score (IL34-FS), interleukin-34 (IL-34), macrophage-colony stimulating factor (M-CSF), soluble CD163 (sCD163), hyaluronic acid, type IV collagen 7s, aspartate transaminase (AST)-to-platelet ratio index (APRI), FIB-4 index, and NAFLD fibrosis score (NFS) as a diagnostic marker of liver fibrosis are shown for all stages. ROC curves for the diagnosis of each stage were as follows: (**A–C**) significant fibrosis (≥Stage 2), (**D–F**) advanced fibrosis (≥Stage 3), (**G–I**) liver cirrhosis (Stage 4). ROC curves were shown as follows: (**A**,**D**,**G**) IL34-FS, IL-34, M-CSF, and sCD163, (**B**,**E**,**H**) IL34-FS, macrophage inflammatory protein-3 alpha/C-C motif chemokine ligand 20 (MIP-3α/CCL20), hyaluronic acid, and type IV collagen 7s, (**C**,**F**,**I**) IL34-FS, APRI, FIB-4 index, and NFS.

In order to establish a better predictive model for the diagnosis of fibrosis stage, we performed multivariate regression analysis using factors which were associated with significant fibrosis, advanced fibrosis, or liver cirrhosis in multivariate analyses. In this study, IL-34, type IV collagen 7s, age were independent markers of fibrosis (Table 2 and Supplementary Table S2–3). We therefore established the predictive model for patients with NAFLD using such factors. IL-34 based fibrosis score (IL34-FS) = 0.0387*IL-34 (pg/ml) + 0.3623*type IV collagen 7s (ng/ml) + 0.0184*age (years)–1.1850. The AUC, sensitivity, and specificity of IL34-FS were, respectively, 0.86, 75.2%, and 85.0% for significant fibrosis (≥Stage 2); 0.88, 81.7%, and 79.4% for advanced fibrosis (≥Stage 3); 0.91, 83.3%, and 85.6% for liver cirrhosis (Stage 4) (Table 4). IL34-FS was significantly superior to the other biomarkers and non-invasive indexes, including IL-34 or type IV collagen 7s alone, among all fibrosis stages (Fig. 4 and Table 4). Using IL34-FS, we were able to estimate liver fibrosis in patients with NAFLD from significant fibrosis to liver cirrhosis.

## Discussion

In this study, we identified serum IL-34 as a marker of liver fibrosis by comprehensive analysis of cytokines, chemokines, and hematopoietic factors. For all fibrosis stages, IL-34 as a diagnostic of fibrosis was superior to the other macrophage-related factors, such as M-CSF and sCD163. With respect to hepatic lipid accumulation, multiple hits are required to advance the stage of NAFLD, or fibrogenesis. Genetic backbone and environmental factors are also reported to be involved^9^. From the perspective of metabolic disorder, oxidative stress and insulin resistance arguably play substantial roles not only in fibrosis but also in carcinogenesis^26^,^27^. In addition, cumulative research evidence indicates that the interaction of macrophages and HSCs underlies the development from simple steatosis to NASH^28^. Therefore, it is rational to consider that some macrophage-related factors are instrumental instigators in the pathogenesis of NASH and fibrogenesis. We thus focused on IL-34 and M-CSF, and compared their diagnostic performance with sCD163 and other well-established fibrosis clinical parameters and fibrosis scores such as hyaluronic acid, type IV collagen 7s, APRI, FIB-4 index, and NFS.

The reasons for the close association of IL-34 with fibrosis in NAFLD, as we found in this study, remain largely unknown. In human adipose tissue, IL-34 is produced from adipocytes in response to pro-inflammatory cytokines, such as TNF-α or IL-1β^29^. Alternatively, Preisser *et al*. recently reported that IL-34 is released from HCV-infected dying hepatocytes and that pro-inflammatory cytokines enhance expression of IL-34 not in hepatocytes, but also in HSCs and fibroblasts^20^. In this study, pro-inflammatory cytokines, including TNF-α and IL-1β, increased in patients with NAFLD compared with those in HVs (data not shown). We showed that IL-34 was mainly expressed on liver fibroblasts (Fig. 3 and Supplementary Fig. S3A,D), the expression of which in liver fibroblasts increased in response to TNF-α (Supplementary Fig. S3E). These data suggest that the enhanced production of fibroblast-derived IL-34 by TNF-α might be one of the contributing factors to the increase of serum IL-34 levels in patients with NAFLD.

The bioactivity of IL-34 in patients with NAFLD is another clinical question to be answered. Other investigators reported that pro-fibrogenic macrophages were generated in the presence of IL-34 *in vitro*, which were capable of producing transforming growth factor-β (TGF-β), platelet-derived growth factor (PDGF)^20^. In this study, IL-34-induced macrophages were able to express MIP-3α/CCL20 (data not shown). Such factors promoted HSC to synthesize type-I collagen^20^,^30^. In a mice model of ischemia and reperfusion kidney injury, IL-34 knock-out mice were reported to have less kidney fibrosis than the wild-type^31^. These observations support the possibility that IL-34 is not only an indicator but also a driver of fibrosis in macrophage-related fibrotic diseases.

Here we propose a predictive model of liver fibrosis based on IL34-FS consisting of serum IL-34, type IV collagen 7s and age, by multiple regression analyses. This predictive model was superior as a marker of fibrosis to other models for all fibrosis stages (Tables 3 and 4 and Fig. 4). IL34-FS may be clinically applicable, because it consists of factors readily measurable and its formulation is simple.

Several limitations could be raised with respect to this study. The usefulness of IL-34 as a marker of fibrosis in other types of liver disease is yet to be proven. It is rational to consider that mutual activation of macrophages and HSC could be a common liver fibrogenesis mechanism, regardless of the etiology of liver disease^15^,^16^,^17^. We showed that IL-34 was increased in sera of HCV patients with advanced liver fibrosis. However, because the size of HCV cohort was small, we were not able to confirm the validity of IL-34 as a diagnostic marker for liver fibrosis in HCV patients. Therefore, further investigation is needed to decide whether IL-34 is a feasible marker of liver fibrosis caused by other etiologies, such as viral hepatitis or autoimmunity. Another limitation is the lack of patients with HCC in this cohort. We were not able to assess the predictive value of IL-34 for HCC occurrence in NAFLD patients. Zhou *et al*. recently reported that the expression levels of IL-34 were associated with overall survival and tumor recurrence rates of HCC^32^. Prospective and longitudinal studies are warranted to elucidate this important clinical issue.

In summary, we showed that IL-34 can serve as an independent marker of liver fibrosis in NAFLD patients, the expression of which is mainly on liver fibroblasts. A novel diagnostic marker IL34-FS, which includes the factors of serum IL-34, type IV collagen 7s and age, could be feasible for the diagnosis of liver fibrosis in patients with NAFLD.

## Materials and Methods

### Subjects

We enrolled 197 NAFLD patients, who were followed at the National Center for Global Health and Medicine, Kohnodai Hospital, and Center Hospital, Nippon Medical School Chiba Hokusoh Hospital, Hiroshima University Hospital, Aichi Medical University Hospital, Kurume University School of Medicine, and Ehime University Graduate School of Medicine.

The diagnosis of NAFLD was based on the liver biopsy specimen showing steatosis (≥5% of hepatocytes containing fat droplets) and the exclusion of other causes of liver disease, such as viral hepatitis, alcoholic liver disease (quantity of ethanol intake >20 g/day for women and 30 g/day for men), drug-induced, or autoimmune liver diseases. No concomitant diseases or conditions causing secondary steatohepatitis, such as endocrine disorders, primary dyslipidemia or malnutrition, were confirmed in the subjects. As disease controls, we enrolled 38 patients with chronic HCV infection. We examined 20 HVs who were negative for HBs-antigen (Ag), HCV-Ab and anti-human immunodeficiency virus (anti-HIV) Ab/Ag and had no apparent history of liver disease or malignancies. The study protocol conformed to the ethical guidelines of human clinical research established by the Japanese Ministry of Health, Labor and Welfare and was approved by the ethics committee in all of the above-mentioned facilities. Written informed consent was obtained from all patients at their enrollment.

### Histological diagnosis

All patients with NAFLD or HCV infection enrolled in this study underwent liver biopsy for the purpose of diagnosis. Tissue specimens of NAFLD patients obtained from National Center for Global Health and Medicine, Kohnodai Hospital, and Center Hospital, Hiroshima University Hospital, Aichi Medical University Hospital, Kurume University School of Medicine, and Ehime University Graduate School of Medicine were collected at the central facility and were subjected to histological diagnosis by three expert pathologists. They independently assessed the stages of fibrosis in NAFLD patients according to Brunt’s criteria^33^. The final diagnosis was based on comparison of their observatory findings if the initial diagnoses were discordant. Histological diagnoses of HCV-infected patients were determined from the METAVIR scores by expert pathologists at each facility^34^.

### Biochemical analysis and calculation of fibrosis indices

Serum samples were collected from all patients before liver biopsy. The indices of APRI, FIB-4 index, and NFS were calculated as reported previously^35^,^36^,^37^.

### Cytokine assay

IL-34, M-CSF, and sCD163 were quantified using enzyme-linked immunosorbent assay kits (R&D Systems, Minneapolis, MN). We used Bio-Plex Pro^TM^ Human Chemokine Panel (40 plex) (Bio-Rad, Hercules, CA) for multiplex assays of the sera.

### Immunohistochemistry

Frozen liver specimens were obtained from surgical resections of non-cancerous liver from patients with NAFLD or HCV infection. The 5-μm serial sections were incubated with the following Abs: mouse anti-human IL-34 monoclonal Ab (1:200, clone 1D12, Abcam, Cambridge, MA) and mouse anti-human α–SMA Ab (1:100, clone 1A4, Sigma-Aldrich, St. Louis, MO), and subsequently with secondary goat anti-mouse Ab (Dako, Carpinteria, CA). Hematoxylin and eosin (H&E) stains were used for recognizing various cell types.

### Immunofluorescence staining

The sections obtained as described above were incubated with the following Abs by detection with a polymeric labeling 2 step method as described^38^: mouse anti-human IL-34 monoclonal Ab (1:200, clone 1D12, Abcam, Cambridge, MA) with secondary goat anti-mouse Alexa Fluor 488 (Invitrogen, Carlsbad, CA) Ab, and rabbit anti-human α-SMA monoclonal Ab conjugated with Alexa Fluor 647 (1:100, clone E184, Abcam, Cambridge, MA) or rabbit anti-human CD68 polyclonal Ab (1:100, clone H-255, Santa Cruz Biotechnology, Santa Cruz, CA) with secondary donkey anti-rabbit Alexa Fluor 647 (Abcam, Cambridge, MA) Ab. Cell nuclei were counterstained with DAPI-Fluoro-KEEPER (Nacalai Tesque, Kyoto, Japan). The stained specimens were analyzed by fluorescence microscopy (Model BZ-9000; Keyence, Osaka, Japan).

### Primary human fibroblasts

Human primary fibroblasts were obtained from surgical resections of non-cancerous liver from NAFLD patients. The resected liver specimens were minced with a scalpel in a culture dish and enzymatically dissociated in 1 mg/ml collagenase (Sigma-Aldrich, St. Louis, MO) at 37 °C for 1 hour with frequent shaking. Cells were plated on a 100 mm dish in Dulbecco’s modified Eagle’s medium (DMEM) supplemented with 10% fetal bovine serum (FBS). Cells from passages 3–10 were used for all experiments. We confirmed that all our primary human fibroblasts were positive for α-SMA, vimentin and negative for Hep Par 1 (data not shown).

### Quantitative reverse-transcriptase polymerase chain reaction analysis

Total RNA was extracted with ISOGEN (Nippon Gene, Tokyo, Japan) and cDNA was generated using transcriptor universal cDNA master (Roche Diagnostics GmbH, Mannheim, Germany). The expression levels of mRNA were quantified by quantitative reverse-transcriptase polymerase chain reaction (qRT-PCR) using commercial primer-probe pairs (Applied Biosystems, Foster City, CA).

### Statistical analysis

The differences between two groups were assessed by Mann-Whitney U-test. Multiple comparisons among more than two groups were analyzed by Kruskal-Wallis and Dunn’s multiple tests. The correlation between two groups was assessed by Spearman’s analysis. Univariate and multivariate analyses were performed by the logistic regression model. The predictive model for liver fibrosis was established using multiple regression analyses. Statistical analyses were performed with Graph Pad Prism software (version 6; Graph Pad Prism, San Diego, CA), SPSS statistical software (version 22.0; SPSS, Chicago, IL), and Microsoft Excel (version 2015).

This research was supported by a grant from the National Center for Global Health and Medicine (26-shi-109)) and by the Research Program on Hepatitis from Japan Agency for Medical Research and development, AMED (15fk0210034h0001).

## Additional Information

**How to cite this article**: Shoji, H. *et al*. Interleukin-34 as a fibroblast-derived marker of liver fibrosis in patients with non-alcoholic fatty liver disease. *Sci. Rep.* **6**, 28814; doi: 10.1038/srep28814 (2016).

## Supplementary Material

Supplementary Information

## Figures and Tables

**Table 1:** Clinical Backgrounds of Subjects in Each Stage of Fibrosis.

	Stage 0–1	Stage 2	Stage 3	Stage 4	All
	(n = 80)	(n = 46)	(n = 41)	(n = 30)	(n = 197)
Male/female	47/33	19/27	14/27	9/21	89/108
Age (year)	50 (22–75)	61 (22–81)	64 (31–83)	66 (47–80)	60 (22–83)
BMI (kg/m^2^)	27.5 (20.8–44.8)	27.1 (20.8–37.4)	28.0 (19.5–41.5)	27.9 (20.1–38.6)	27.5 (19.5–44.8)
Platelet (×10^4^/mm^3^)	20.7 (7.8–32.9)	20.4 (10.4–40.5)	19.5 (8.2–35.1)	13.1 (6.5–30.7)	19.8 (6.5–40.5)
PT (%)	103 (67–147)	96 (74–140)	89 (72–112)	79 (54–123)	95 (54–147)
Total bilirubin (mg/dL)	0.8 (0.2–2.7)	0.8 (0.3–1.4)	0.8 (0.4–2.4)	0.9 (0.3–1.6)	0.8 (0.2–2.7)
AST (IU/L)	41 (15–147)	53 (19–245)	56 (14–186)	54 (27–185)	48 (14–245)
ALT (IU/L)	75 (14–277)	70 (19–539)	67 (13–318)	53 (8–194)	66 (8–539)
Cholesterol (mg/dL)	198 (97–335)	202 (102–378)	185 (117–331)	177 (113–368)	193 (97–378)
Albumin (g/dL)	4.3 (3.2–5.3)	4.3 (3.6–5.4)	4.3 (2.6–4.9)	4.0 (3.4–4.7)	4.3 (2.6–5.4)
Hyaluronic acid (ng/ml)	28 (8–210)	43 (12–477)	65 (10–420)	139 (29–480)	45 (8–480)
Type IV collagen 7s (ng/ml)	4.1 (1.9–8.3)	5.1 (2.5–9.3)	6.0 (3.8–8.9)	7.8 (5–15)	4.9 (1.9–15)
APRI	0.57 (0.23–2.36)	0.77 (0.25–2.86)	1.14 (0.27–3.41)	1.19 (0.27–5.50)	0.79 (0.23–5.50)
FIB-4 index	1.11 (0.40–5.64)	1.58 (0.55–4.98)	2.48 (0.70–8.02)	3.95 (0.82–10.1)	1.7 (0.40–10.1)
NFS	−2.37 (−4.20–2.47)	−1.01 (−4.47–1.95)	−0.43 (−3.03–1.80)	0.54 (−1.76–4.34)	−0.96 (−4.47–4.34)

The values are expressed as median (range). BMI, body mass index; PT, prothrombin time; AST, aspartate transaminase; ALT, alanine transaminase; APRI, aspartate transaminase (AST)-to-platelet ratio index; NFS, NAFLD fibrosis score.

**Table 2:** Variable Parameters Associated with Liver Cirrhosis (Stage 4) According to Univariate and Multivariate Analyses.

	Liver Cirrhosis (Stage 4)					
	UVA			MVA		
	OR	95% CI	p value	OR	95% CI	p value
Female: 0, Male: 1	0.466	0.202–1.077	0.074			
Age	1.057	1.021–1.095	**0.002**	1.031	0.973–1.094	0.300
BMI (kg/m^2^)	1.040	0.961–1.126	0.332			
Platelet	0.845	0.782–0.913	**<0.001**	0.906	0.820–1.000	0.051
PT (%)	0.937	0.907–0.969	**<0.001**	1.001	0.965–1.037	0.972
Total bilirubin	0.909	0.445–1.855	0.792			
AST	1.003	0.993–1.013	0.588			
ALT	0.992	0.983–1.001	0.084			
Cholesterol	0.988	0.978–0.998	**0.023**	1.001	0.989–1.013	0.905
Albumin	0.144	0.050–0.413	**<0.001**	0.436	0.111–1.721	0.236
Hyaluronic acid	1.011	1.006–1.015	**<0.001**	1.001	0.995–1.007	0.768
Type IV collagen 7S	2.367	1.766–3.174	**<0.001**	1.910	1.264–2.888	**0.002**
IL-34	1.399	1.234–1.586	**<0.001**	1.233	1.060–1.433	**0.006**
M-CSF	1.001	1.000–1.002	**0.024**	1.000	0.999–1.001	0.498
sCD163	1.002	1.001–1.003	**<0.001**	1.000	0.999–1.001	0.889
MIP-3α/CCL20	1.006	0.996–1.016	0.264			
APRI	2.587	1.575–4.250	**<0.001**			
FIB-4 index	1.923	1.500–2.466	**<0.001**			
NFS	2.476	1.734–3.535	**<0.001**			

UVA, univariate analysis; MVA, multivariate analysis; OR, odds ratio; CI, confidence interval; IL-34, interleukin-34; M-CSF, macrophage colony-stimulating factor; sCD163, soluble CD163; MIP-3α/CCL20, macrophage inflammatory protein-3 alpha/C-C motif chemokine ligand 20. BMI, PT, AST, ALT, APRI, NFS, see Table 1.

**Table 3:** Comparison of Performance of Biomarkers and Fibrosis Scores as a Diagnostic of Liver Fibrosis by ROC Analyses.

	IL-34	M-CSF	sCD163	MIP-3α/CCL20	Hyaluronic acid	Type IV collagen 7s	APRI	FIB-4 index	NFS
Significant Fibrosis (≥Stage 2)									
AUC	0.68	0.66	0.67	0.62	0.77	0.85	0.70	0.76	0.75
Cutoff value	4.18	582	622	6.08	38	4.9	0.752	1.37	−1.10
Sensitivity (%)	70.9	63.2	65.8	58.1	75.2	76.1	66.7	77.8	70.1
Specificity (%)	63.7	67.5	65.0	63.7	75.0	85.0	66.2	67.5	68.7
PPV (%)	74.1	74.0	73.3	70.1	81.5	88.1	74.3	77.8	76.6
NPV (%)	59.9	55.6	56.5	51.0	67.4	70.9	57.6	67.5	61.1
Predictive accuracy (%)	68.0	64.9	65.5	60.4	75.1	79.7	66.5	73.6	69.5
Advanced Fibrosis (≥Stage 3)									
AUC	0.76	0.62	0.68	0.62	0.80	0.86	0.72	0.80	0.80
Cutoff value	5.33	642	708	7.43	48	5.1	0.886	2.24	−0.533
Sensitivity (%)	64.8	59.2	67.6	54.9	83.1	81.7	67.6	71.8	69
Specificity (%)	78.6	66.7	67.5	65.9	71.4	74.6	69.0	78.6	77.8
PPV (%)	63.0	50.0	54.0	47.6	62.1	64.4	55.1	65.4	63.7
NPV (%)	79.8	74.4	78.7	72.2	88.2	87.9	79.1	83.2	81.7
Predictive accuracy (%)	73.6	64.0	67.5	61.9	75.6	77.2	68.5	76.1	74.6
Liver Cirrhosis (Stage 4)									
AUC	0.87	0.63	0.77	0.67	0.86	0.88	0.71	0.83	0.83
Cutoff value	6.27	667	723	6.08	84	6.3	0.811	2.48	−0.014
Sensitivity (%)	83.3	63.3	83.3	70.0	86.7	83.3	73.3	80.0	80.0
Specificity (%)	80.2	66.5	64.1	54.5	67.2	79.0	56.3	76.0	80.8
PPV (%)	43.0	25.3	29.4	21.6	32.2	41.6	23.2	37.5	42.8
NPV (%)	96.4	91.0	95.5	91.0	96.6	96.3	92.1	95.5	95.7
Predictive accuracy (%)	80.7	66.0	67.0	56.9	70.2	79.7	58.9	76.6	80.7

The optimal cutoff values are determined as those yielding the minimal value for (1 − sensitivity)^2^ + (1 − specificity)^2^. Such values with those sensitivity and specificity values are the closest to the (0, 1) point on the receiver-operating characteristic (ROC). AUC, area under receiver operating characteristic; PPV, positive predictive values; NPV, negative predictive values. IL-34, M-CSF, sCD163, MIP-3α/CCL20, APRI, NFS, see Table 2.

**Table 4:** The ability of IL34-FS as a Diagnosis of Liver Fibrosis by ROC Analyses.

	IL34-FS
Significant Fibrosis (≥Stage 2)	
AUC	0.86
Cutoff value	1.86
Sensitivity (%)	75.2
Specificity (%)	85.0
PPV (%)	88.0
NPV (%)	70.1
Predictive accuracy (%)	79.2
Advanced Fibrosis (≥Stage 3)	
AUC	0.88
Cutoff value	2.02
Sensitivity (%)	81.7
Specificity (%)	79.4
PPV (%)	69.1
NPV (%)	88.5
Predictive accuracy (%)	80.2
Liver Cirrhosis (Stage 4)	
AUC	0.91
Cutoff value	2.67
Sensitivity (%)	83.3
Specificity (%)	85.6
PPV (%)	51.0
NPV (%)	96.6
Predictive accuracy (%)	85.2

The cutoff values, sensitivity, and specificity were determined as described in Table 3. AUC, ROC, PPV, NPV, see Table 3. IL34-FS, interleukin34 based fibrosis score.
