# Supplementary material for: Interleukin-34 as a fibroblast-derived marker of liver fibrosis in patients with non-alcoholic fatty liver disease
Source: Sci Rep. 2016 Jul 1;6:28814. doi: 10.1038/srep28814 (PMC4929441; doi:10.1038/srep28814)

Supplementary information

Interleukin-34 as a fibroblast-derived marker of liver fibrosis in patients with non-alcoholic fatty liver disease

Hirotaka Shoji[1,2], Sachiyo Yoshio[1], Yohei Mano[1], Erina Kumagai[1], Masaya Sugiyama[1], Masaaki Korenaga[1], Taeang Arai[3], Norio Itokawa[3], Masanori Atsukawa[3], Hiroshi Aikata[4], Hideyuki Hyogo[4,5], Kazuaki Chayama[4], Tomohiko Ohashi[6], Kiyoaki Ito[6], Masashi Yoneda[6], Yuichi Nozaki[7], Takumi Kawaguchi[8], Takuji Torimura[8], Masanori Abe[9], Yoichi Hiasa[9], Toshiya Kamiyama[2], Akinobu Taketomi[2], Masashi Mizokami[1] and Tatsuya Kanto[1]*

1 The Research Center for Hepatitis and Immunology, National Center for Global Health and Medicine, Ichikawa, Chiba, Japan

2 Department of Gastroenterological Surgery I, Hokkaido University Graduate School of Medicine, Sapporo, Hokkaido, Japan

3 Division of Gastroenterology, Department of Internal Medicine, Nippon Medical School Chiba Hokusoh Hospital, Inzai, Chiba, Japan

4 Department of Gastroenterology and Metabolism, Applied Life Sciences, Institute of Biomedical & Health Sciences, Hiroshima University, Hiroshima, Hiroshima, Japan

5 Department of Gastroenterology and Hepatology, JA Hiroshima General Hospital, Hatsukaichi, Hiroshima, Japan

6 Division of Gastroenterology, Department of Internal Medicine, Aichi Medical University School of Medicine, Nagakute, Aichi, Japan

7 Department of Gastroenterology, National Center for Global Health and Medicine, Shinjuku, Tokyo, Japan

8 Division of Gastroenterology, Department of Medicine, Kurume University School of Medicine, Kurume, Fukuoka, Japan

9 Department of Gastroenterology and Metabology, Ehime University Graduate School of Medicine, Toon, Ehime, Japan

Corresponding author: Tatsuya Kanto, M.D., Ph.D.

The Research Center for Hepatitis and Immunology, National Center for Global Health and Medicine, 1-7-1, Kohnodai, Ichikawa 272-8516, Japan.

E-mail: kantot@hospk.ncgm.go.jp

Telephone: +81-47-372-3501, Fax: +81-47-375-4766

Supplementary Table S1. List of Cytokines/Chemokines for Multiplexed Analyses

6Ckine/CCL21	Gro-α/CXCL1	IL-6	MIP-1α/CCL3
BCA-1/CXCL13	Gro-β/CXCL2	IL-8/CXCL8	MIP-1δ/CCL15
CTACK/CCL27	I-309/CCL1	IP-10/CXCL10	MIP-3α/CCL20
ENA-78/CXCL5	I-TAC/CXCL11	MCP-1/CCL2	MIP-3β/CCL19
Eotaxin-2/CCL24	IFN-γ	MCP-2/CCL8	MPIF-1/CCL23
Eotaxin-3/CCL26	IL-10	MCP-3/CCL7	SCYB16/CXCL16
Eotaxin/CCL11	IL-16	MCP-4/CCL13	SDF-1α+β/CXCL12
Fractalkine/CX3CL1	IL-1β	MDC/CCL22	TARC/CCL17
GCP-2/CXCL6	IL-2	MIF	TECK/CCL25
GM-CSF	IL-4	MIG/CXCL9	TNF-α

Supplementary Table S2. Variable Parameters Associated With Significant Fibrosis (≥Stage 2) According to Univariate and Multivariate Analyses

	Significant Fibrosis (≥Stage 2)					
	UVA			MVA		
	OR	95% CI	p value	OR	95% CI	p value
Female: 0, Male: 1	0.393	0.219-0.705	**0.002**	2.041	0.792-5.264	0.140
Age	1.054	1.031-1.078	**<0.001**	1.054	1.018-1.091	**0.003**
BMI (kg/m^2)	1.024	0.963-1.089	0.440			
Platelet	0.970	0.928-1.013	0.171	1.089	1.002-1.184	**0.046**
PT (%)	0.945	0.924-0.968	**<0.001**	0.972	0.944-1.002	0.068
Total bilirubin	1.046	0.658-1.664	0.848			
AST	1.020	1.009-1.031	**<0.001**	1.011	0.996-1.026	0.163
ALT	1.002	0.997-1.007	0.421			
Cholesterol	0.996	0.990-1.003	0.237			
Albumin	0.471	0.227-0.977	**0.043**	1.305	0.444-3.836	0.629
Hyaluronic acid	1.017	1.010-1.025	**<0.001**	1.004	0.998-1.011	0.205
Type IV collagen 7S	2.996	2.135-4.204	**<0.001**	2.259	1.447-3.528	**<0.001**
IL-34	1.221	1.096-1.360	**<0.001**	0.978	0.849-1.128	0.763
M-CSF	1.002	1.001-1.003	**<0.001**	1.001	0.999-1.002	0.152
sCD163	1.002	1.001-1.003	**<0.001**	1.000	0.999-1.002	0.735
MIP-3α/CCL20	1.006	0.993-1.019	0.380			
APRI	3.570	1.931-6.601	**<0.001**			
FIB-4 index	2.060	1.535-2.765	**<0.001**			
NFS	1.853	1.483-2.314	**<0.001**			

UVA, univariate analysis; MVA, multivariate analysis; OR, odds ratio; CI, confidence interval

IL-34, M-CSF, sCD163, MIP-3α/CCL20 see Table 2.

BMI, PT, AST, ALT, APRI, NFS see Table 1.

Supplementary Table S3. Variable Parameters Associated with Advanced Fibrosis (≥Stage 3) According to Univariate and Multivariate Analyses

	Advanced Fibrosis (≥Stage 3)					
	UVA			MVA		
	OR	95% CI	p value	OR	95% CI	p value
Female: 0, Male: 1	0.436	0.237-0.800	**0.007**	1.301	0.500−3.38	0.589
Age	1.059	1.033-1.086	**<0.001**	1.040	1.002-1.079	**0.037**
BMI (kg/m^2)	1.024	0.963-1.089	0.443			
Platelet	0.914	0.870-0.961	**<0.001**	0.982	0.910-1.058	0.628
PT (%)	0.932	0.908-0.958	**<0.001**	0.974	0.942-1.007	0.125
Total bilirubin	0.962	0.598-1.547	0.872			
AST	1.011	1.002-1.019	**0.012**	1.006	0.993-1.019	0.403
ALT	0.999	0.994-1.004	0.632			
Cholesterol	0.992	0.985-0.999	**0.029**	0.999	0.990-1.010	0.988
Albumin	0.280	0.129-0.608	**0.001**	0.776	0.260-2.314	0.648
Hyaluronic acid	1.012	1.007-1.017	**<0.001**	1.001	0.995-1.007	0.720
Type IV collagen 7S	2.496	1.928-3.231	**<0.001**	1.940	1.331-2.827	**<0.001**
IL-34	1.335	1.193-1.494	**<0.001**	1.125	0.981-1.289	0.092
M-CSF	1.001	1.000-1.002	**0.003**	1.001	0.999-1.001	0.290
sCD163	1.001	1.001-1.002	**0.001**	0.999	0.998-1.001	0.370
MIP-3α/CCL20	1.006	0.995-1.017	0.278			
APRI	3.294	1.963-5.528	**<0.001**			
FIB-4 index	2.232	1.705-2.921	**<0.001**			
NFS	2.181	1.691-2.813	**<0.001**			

UVA, MVA, OR, CI see Supplementary Table S2

IL-34, M-CSF, sCD163, MIP-3α/CCL20 see Table 2.

BMI, PT, AST, ALT, APRI, NFS see Table 1.

Supplementary Table S4. Clinical Backgrounds of Subjects with HCV Infection

	Stage 0-2 (n = 31)<br>(n = 31)	Stage 3-4 (n = 7)<br>(n = 7)	All (n = 38)<br>(n = 38)
Male/female	16/15	1/6	17/21
Age (year)	57 (27-76)	71(54-88)	61 (27-88)
BMI (kg/m^2)	22.4 (17.0-30.9)	25.2 (15.9-27.7)	22.8 (15.9-30.9)
Platelet (×10^4/mm^3)	17.2 (8.3-34.1)	8.8 (5.2-13.5)	16.4 (5.2-34.1)
PT (%)	97 (76-129)	85 (63-95)	95 (63-129)
Total bilirubin (mg/dL)	0.6 (0.4-1.3)	0.8 (0.6-1.5)	0.7 (0.4-1.5)
AST (IU/L)	32 (17-253)	71 (37-206)	43 (17-253)
ALT (IU/L)	41 (17-362)	56 (31-294)	43 (17-362)
Cholesterol (mg/dL)	169 (120-251)	144 (133-187)	167 (120-251)
Albumin (g/dL)	4.3 (2.9-4.9)	3.7 (3.3-4.4)	4.2 (2.9-4.9)
APRI	0.66 (0.18-2.83)	2.44 (1.38-5.38)	0.81 (0.18-5.38)
FIB-4 index	1.96 (0.55-6.42)	6.69 (4.20-9.80)	2.3 (0.55-9.80)

The values are expressed as median (range). BMI, PT, AST, ALT, APRI see Table 1.

Supplementary Table S5. Comparison of Performance of Biomarkers and Fibrosis Scores as a Diagnostic of Liver Fibrosis in Patients with HCV Infection in ≥Stage 3

Severe Fibrosis (≥Stage 3)	IL-34	M-CSF	sCD163	APRI	FIB-4 index
AUC	0.95	0.75	0.85	0.93	0.98
Cutoff value	6.59	779	1148	1.38	4.20
Sensitivity (%)	100	71.4	85.7	100	100
Specificity (%)	83.9	74.2	74.2	80.6	90.3
PPV (%)	58.3	38.5	42.9	53.8	70.0
NPV (%)	100	92.0	95.8	100	100
Predictive accuracy (%)	86.9	73.7	76.3	84.2	92.1

The cutoff values, sensitivity, and specificity were determined as described in Table 3.

AUC, PPV, NPV, IL-34, M-CSF, sCD163, APRI, see Table 3.

Supplementary Figure legend

Supplementary Figure S1: Serum IL-34 and other markers in patients with HCV infection.

Serum interleukin-34 (IL-34), other biomarkers and the fibrosis scores are shown for healthy volunteers and patients with hepatitis C virus (HCV) infection categorized by the stage of fibrosis (0-2 and 3-4 by METAVIR classification) (**A-E**). IL-34 (**A**), macrophage-colony stimulating factor (M-CSF) (**B**), soluble CD163 (sCD163) (**C**), aspartate transaminase (AST)-to-platelet ratio index (APRI) (**D**), and FIB-4 index (**E**). Receiver-operating characteristic (ROC) curves of IL-34, M-CSF, sCD163, APRI and, FIB-4 index as a diagnostic marker of advanced liver fibrosis (≥Stage 3) are shown (**F-G**). ROC curves were shown as follows: (**F**) IL-34, M-CSF, and sCD163, (**G**) IL-34, APRI, and FIB-4 index.

Box plots are as described in Figure 1.

** $p<0.01$, **** $p<0.0001$ by Mann-Whitney U-test or Kruskal-Wallis test with Dunn’s multiple comparison test

HV, healthy volunteer; 0-2, Stage 0-2 (Brunt’s criteria); 3-4, Stage 3-4.

Supplementary Figure S2: Immunofluorescence staining of IL-34 and CD68 on liver specimens obtained from the patient with NAFLD.

Immunofluorescence staining on frozen liver specimens from the patient with NAFLD was shown (x200, green, IL-34; red, CD68; blue, DAPI).

Supplementary Figure S3: The expression of IL-34 in primary fibroblasts obtained from patients with NAFLD.

Primary fibroblasts obtained from the cirrhotic patient with non-alcoholic fatty liver disease (NAFLD) [#3 NAFLD-liver cirrhosis (LC)] were labeled with anti-human interleukin-34 (IL-34) monoclonal and secondary antibody (Ab) (**A, left**) and with only secondary Ab (**A, right**). Immunofluorescence staining on primary fibroblast was shown (**B**) (x400, green, IL-34; red, α-SMA; blue, DAPI). Primary fibroblasts were isolated from three surgically resected liver specimens of NAFLD patients with chronic hepatitis [#1 NAFLD-chronic hepatitis (CH)] and liver cirrhosis (#2 and #3 NAFLD-LC). Huh7 (hepatocellular carcinoma cell line), LX-2 (hepatic stellate cell line) and primary fibroblast cells ($3x10^4$ cells/ml/well in 24-well plates) were cultured in fetal bovine serum (FBS)-free culture medium for 24 hours (starvation). After starvation, the cells were incubated for 24 hours and the cells and supernatant were

harvested. The expression levels of IL-34 mRNA were examined by quantitative reverse-transcriptase polymerase chain reaction (qRT-PCR) (**C**). The levels of IL-34 in the culture supernatant were quantified by enzyme-linked immunosorbent assay (ELISA) (**D**). Huh7, LX-2, and primary fibroblasts were cultured with or without recombinant tumor necrosis factor alpha (TNF-α) (10 ng/ml) for 24 h. The levels of IL-34 mRNA were examined by qRT-PCR (**E**).

rhTNF-α, recombinant TNF-α; n.d.,not detected

** $p<0.01$, *** $p<0.001$, **** $p<0.0001$ by Kruskal-Wallis test with Dunn's multiple comparison test

Supplementary Figure S4: Immunostaining of IL-34 in cirrhotic liver tissue obtained from the patients with HCV infection.

Noncancerous liver specimen was obtained from patients with HCV infection (liver cirrhosis/ Stage 4). Liver specimen was labeled with mouse anti-human IL-34 monoclonal and secondary goat anti-mouse antibody.

Supplementary Figure S1

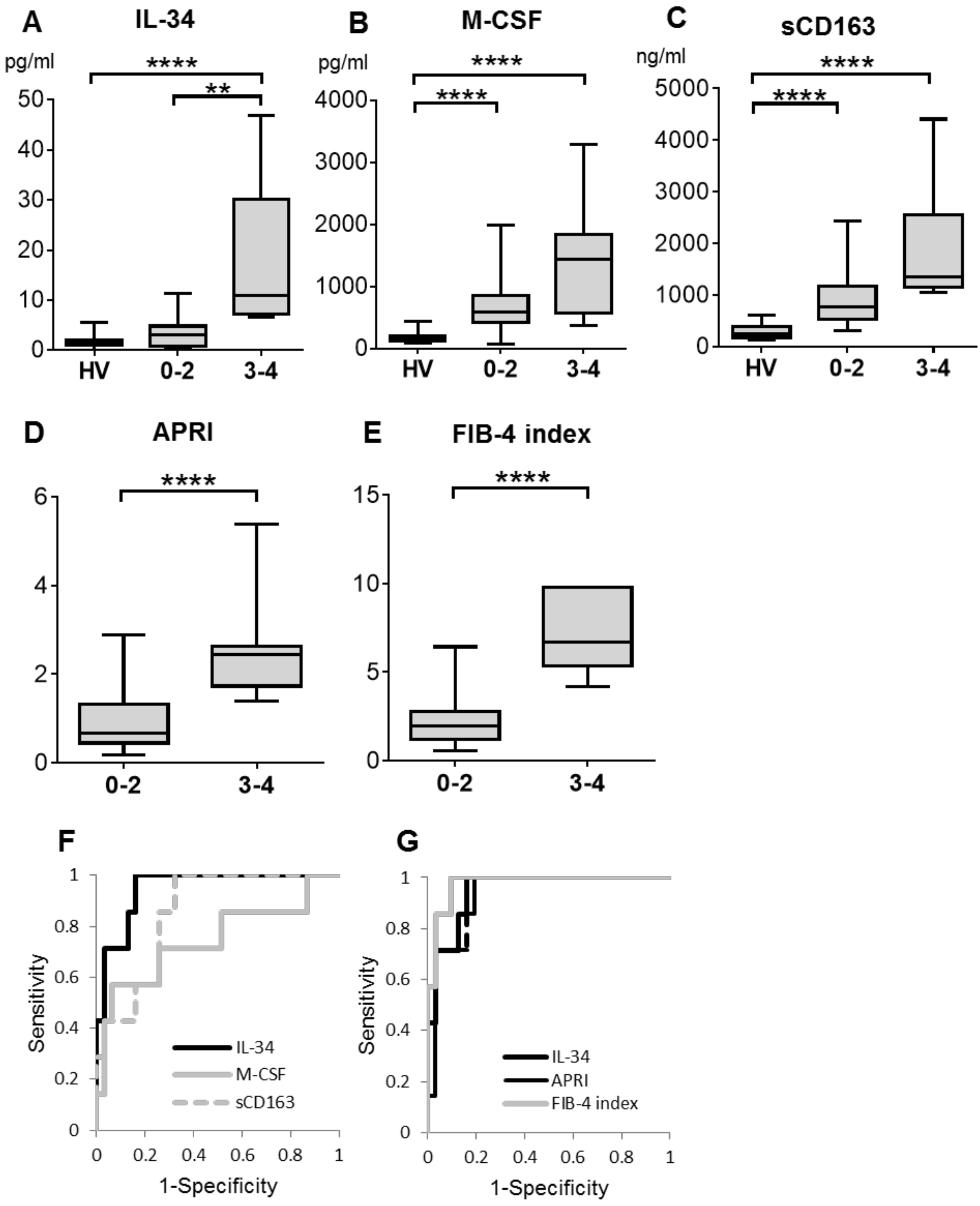

Supplementary Figure S2

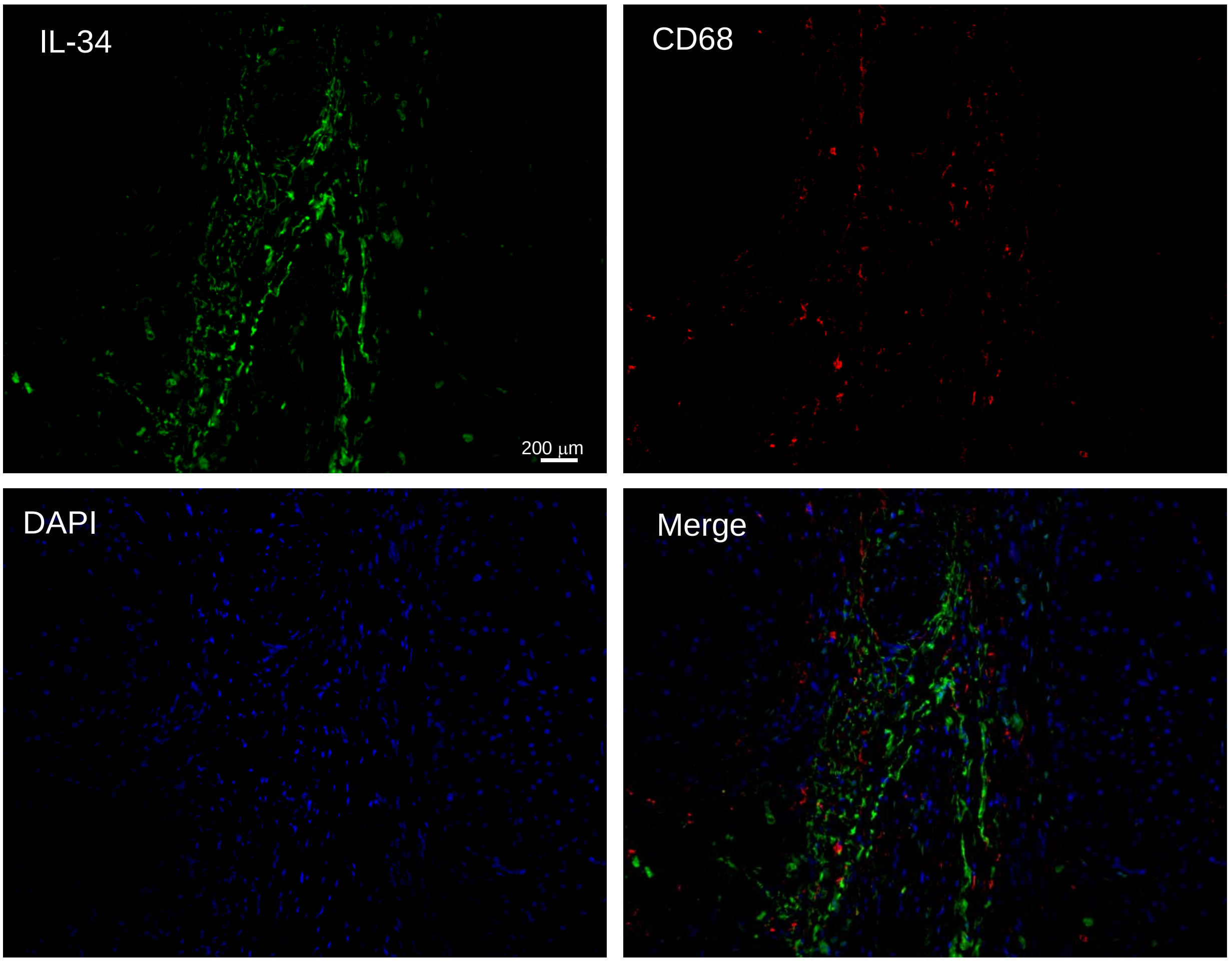

Supplementary Figure S3

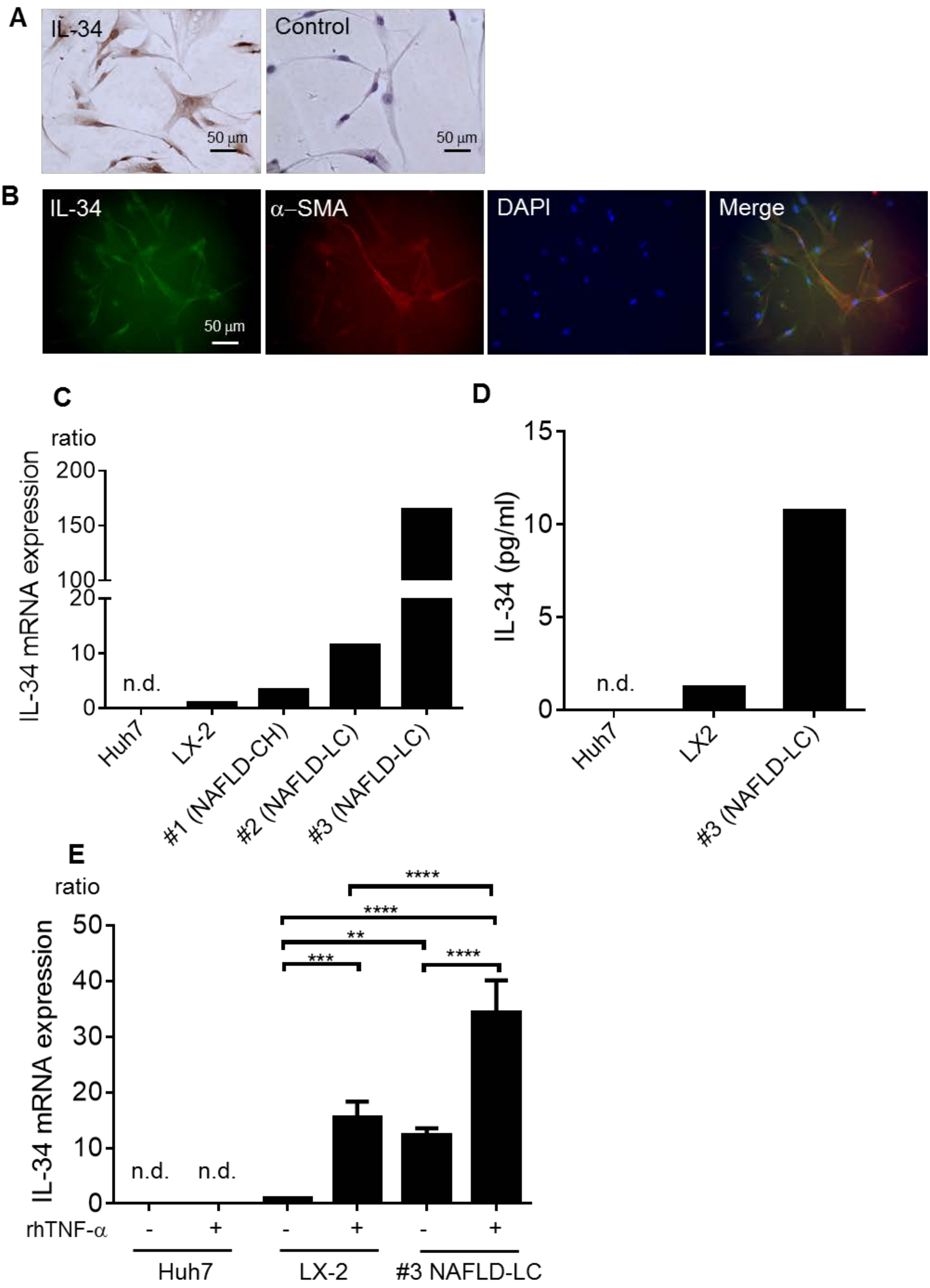

Supplementary Figure S4

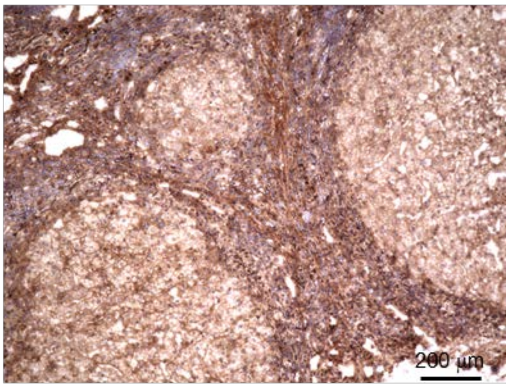

Supplement: Supplementary Information [file srep28814-s1.pdf]
